# Overview of national health reporting in the EU and quality criteria for public health reports – results of the Joint Action InfAct

**DOI:** 10.1186/s13690-021-00753-7

**Published:** 2021-12-22

**Authors:** Martin Thissen, Stefanie Seeling, Peter Achterberg, Angela Fehr, Luigi Palmieri, Mariken J. Tijhuis, Brigid Unim, Thomas Ziese

**Affiliations:** 1 Unit 24 – Health Reporting, Department of Epidemiology and Health Monitoring, Robert Koch Institute, General-Pape-Str. 62-66, 12101 Berlin, Germany; 2 Centre for Health Knowledge Integration, National Institute for Public Health and the Environment (RIVM), Bilthoven, The Netherlands; 3 ZIG 1 – Information Centre for International Health Protection (INIG), Centre for International Health Protection (ZIG), Robert Koch Institute, Nordufer 20, 13353 Berlin, Germany; 4 Department of Cardiovascular, Endocrine-metabolic Diseases and Aging, Istituto Superiore di Sanità, Rome, Italy

**Keywords:** Health reporting, Public health report, Health information, Recommendations, Quality criteria, Reporting format, Target group, Dissemination, Inequalities

## Abstract

**Background:**

Health reporting shall provide up-to-date health-related data to inform policy-makers, researchers and the public. To this end, health reporting formats should be tailored to the needs and competencies of the target groups and provide comparable and high-quality information. Within the Joint Action on Health Information ‘InfAct’, we aimed at gaining an overview of health reporting practices in the EU Member States and associated countries, and developed quality criteria for the preparation of public health reports. The results are intended to facilitate making health information adequately available while reducing inequalities in health reporting across the EU.

**Methods:**

A web-based desk research was conducted among EU Member States and associated countries to generate an overview of different formats of national health reporting and their respective target groups. To identify possible quality criteria for public health reports, an exploratory literature review was performed and earlier projects were analysed. The final set of criteria was developed in exchange with experts from the InfAct consortium.

**Results:**

The web-based desk research showed that public health reports are the most frequently used format across countries (94%), most often addressed to scientists and researchers (51%), politicians and decision-makers (41%). However, across all reporting formats, the general public is the most frequently addressed target group. With regards to quality criteria for public health reports, the literature review has yielded few results. Therefore, two earlier projects served as main sources: the ‘Evaluation of National and Regional Public Health Reports’ and the guideline ‘Good Practice in Health Reporting‘from Germany. In collaboration with experts, quality criteria were identified and grouped into eight categories, ranging from topic selection to presentation of results, and compiled in a checklist for easy reference.

**Conclusion:**

Health reporting practices in the EU are heterogeneous across Member States. The assembled quality criteria are intended to facilitate the preparation, dissemination and access to better comparable high-quality public health reports as a basis for evidence-based decision-making. A comprehensive conceptual and integrative approach that incorporates the policy perspective would be useful to investigate which dissemination strategies are the most suitable for specific requirements of the targeted groups.

## Background

Health reporting shall provide up-to-date data and descriptions of the population‘s health status and its determinants, and identify areas where action is needed in health care, health protection, health promotion and disease prevention. Establishing an information or discussion base for health policy is a key objective of health reporting (‘data for action’) [1]. Therefore, policy-makers are an important target group of health reporting, next to scientists and researchers, health care providers, the media as well as the general public [2]. Yet there is often a gap in public health science between gaining new knowledge and its translation into practice and policy.

National health reporting has to meet a number of important requirements. The process of developing health information (HI) for national health reporting can be described as ranging from the selection of topics to the delivery of health reports that are to be based on carefully defined indicators with underlying quality data. Quality standards are linked to selected steps in this development process, such as data availability, indicator development and preparing as well as disseminating health reports. Equally, structured and transparent processes should be applied to selecting topics for national health reporting [3].

Depending on the needs and competencies of the addressees, it is important to develop dissemination strategies, including suitable formats [4], which provide an adequate form of communication to share public health messages with a desired audience [5]. Particularly in the area of formats and communication channels, digitalisation opens up new possibilities for the visualisation and processing of data [6]. In addition to printed formats, online formats like websites, dashboards or social media are also becoming increasingly important [7]. This development is further fueled by the COVID-19 pandemic, which highlighted an urgent need for up-to-date data and information.

Health reports cover a broad spectrum of subjects, ranging from diseases, risk and protective factors to subjective well-being and health-related quality of life, utilisation of healthcare services as well as the structures and costs of healthcare systems. In general, health reports can be divided into two main types (considered here as two different formats): public health reports and health system performance assessment (HSPA) reports. Subcategories are among others ‘topical‘comprehensive reports on, for instance, infectious diseases, chronic diseases or lifestyle factors, and on the other hand ‘thematic‘reports focusing on specific subjects or population groups, for example, about health prevention in children or health care of older persons [1].

Heterogeneity of health reporting practices across EU MS as well as occasional language barriers cause difficulties in facilitating access to EU-comparable health information [1]. To tackle inequalities in health reporting across the EU and to facilitate making health information adequately accessible and available is the aim of the InfAct project, that builds towards a sustainable infrastructure on EU health information [8]. This paper presents the results of the project activities that aimed:

1. to prepare a comprehensive overview of the different formats of national health reporting for the dissemination of health information and their target groups,
2. to facilitate desirable quality criteria for preparing EU-comparable public health reports.

The results of two earlier projects were of particular relevance for the development of the quality criteria: the research project ‘Evaluation of National and Regional Public Health Reports’ (Eva PHR) [9] and the guideline ‘Good Practice in Health Reporting‘from Germany [10] .

## Methods

The methodology is described in two sections corresponding to the two components of the research activities: The web-based desk research to provide an overview of health reporting practices in EU MS and associated countries, and the development of quality criteria for public health reports based on the results of earlier projects and exchange with experts.

### Web-based desk research on national health reporting in the EU

A web-based desk research was conducted among EU MS and associated countries on national health reporting formats and their respective target groups. A detailed description of the methodology has been published on the InfAct website [1]. In this article, we will therefore limit ourselves to a brief summary of the most relevant aspects.

A method paper for an explorative search strategy on the status of health reporting in the EU MS and associated countries was drafted and circulated among experts from the InfAct consortium for review and approval. A pre-test of the search strategy was conducted in the federal states of Germany as well as in selected EU MS, and an analysis plan was drafted. Potential sources for national health reporting, including Public Health Institutes, Ministries of Health and Statistical Institutes were identified using the list of members of the International Association of National Public Health Institutes (IANPHI). In case of missing information from IANPHI countries or for countries that are not members of the IANPHI, a Google search for potential sources was carried out. On the websites of these institutes and ministries, an explorative search was executed manually for relevant health reporting formats and their target groups. Since not all countries provide an English translation of national health reports or national-language websites of their Public Health Institutes, Ministries of Health or Statistical Institutes, the reports and websites were translated into English using the Google Translate tool. Subsequently, a Google keyword search was conducted with various combinations of search terms, followed by a search on Google scholar and an exploratory literature review on PubMed/ Embase using the same keywords to close further possible gaps:

- ‘Health reporting‘ OR ‘health reports’ OR ‘healthcare‘+ [country].
- ‘Public health reporting‘ OR ‘public health reports’ + [country].
- ‘Health reporting‘ OR ‘health reports’ OR ‘healthcare‘+ ‘strategy‘+ [country].
- ‘Health reporting‘ OR ‘health reports’ OR ‘healthcare‘+ ‘formats‘+ [country].
- ‘Health reporting‘ OR ‘health reports’ OR ‘healthcare‘+ ‘indicators‘+ [country].
- ‘Health reporting‘ OR ‘health reports’ OR ‘healthcare‘+ ‘target group‘+ [country].
- ‘Health reporting‘ OR ‘health reports’ OR ‘healthcare‘+ ‘good practice‘+ [country].
- ‘Health reporting‘ OR ‘health reports’ OR ‘healthcare‘+ ‘recommendations‘+ [country].
- ‘Health reporting‘ OR ‘health reports’ OR ‘healthcare‘+ ‘guidelines‘+ [country].

To categorise the findings of the web-based desk research, a list of health reporting formats including their description was established on the basis of the exploratory literature review on PubMed/ Embase and input of experts from the InfAct consortium. Table 1 shows the twelve formats that were considered in the analysis.

**Table 1:** Health reporting formats

Format	Description	Pages
Public Health Report	Comprehensive and detailed description of a variety of topics	~ 50–200
Health System Performance Assessment (HSPA) Report	Country-specific process of monitoring, evaluating, communicating and reviewing the achievement of high-level health system goals based on health system strategies	~ 50–200
Short Report	Topic-specific presentation of results and interpretation	~ 10–30
Fact Sheet	Standardised presentation of circumscribed analyses	~ 1–10
Scientific Publication	Publication of specific topics relevant to science	~ 2–10
Scientific Journal	Publisher of his own scientific journal	~ 20–100
Flyer/ Brochure/ Leaflet	Compressed and simplified display of summarised public health information	~ 2–3
Website	All websites that provide health information	–
Statistical online-database	Provision of collected data for own analyses	–
Video	Visualised simplified and comprehensible dissemination of health information	–
Social Media	Dissemination of health information via Facebook, Twitter, Instagram	–
Workshop/ Seminar	Face-to-face communication; documentation of workshop or seminar	–

For the analysis, eight categories of target groups were defined and were regarded as relevant addressees of health reporting according to the experts’ assessment. The categorization builds upon the four central addressees named in the World Health Organization’s (WHO) framework for the implementation of surveillance of non-communicable diseases: health care providers (e.g. physicians, nurses), politicians, decision-makers in the health care system and the general public [11]. For the purpose of this research, they were regrouped and supplemented by results from other sources [2, 5–7, 9, 12, 13] as well as input of the experts in the field:

- Politicians/ Decision-makers
- Health care providers
- Scientists/ Researchers
- Health educators
- General public
- Patients
- Media/ Press
- Civil society groups and community organisations

The findings of the web-based desk research were analysed according to a qualitative content analysis, described by Mayring [14], to assign the results to the formats and target groups. In a further step, the univariate analysis of the research results followed, before cross-comparisons between the categories were carried out to identify which target groups are addressed by the different health reporting formats [1].

### Development of quality criteria for public health reports

An exploratory literature review on PubMed/ Embase and exchange with experts in the field showed that there is only a limited number of publications touching on quality criteria for public health reports. However, two earlier projects have provided valuable information – the project ‘Evaluation of National and Regional Public Health Reports’ (Eva PHR) [9] and the German guideline ‘Good Practice in Health Reporting‘ [10]. The quality criteria for preparing public health reports could therefore mainly be identified on the basis of these two sources, supplemented by findings from the literature [2, 4, 6, 7, 15–27] in the field of public health and in general research communication (Table 2).

**Table 2:** Selected references

Authors	Year	Title
Bernhardt JM [15]	2004	Communication at the core of effective public health
Bou-Karroum L, El-Jardali F, Hemadi N et al. [16]	2017	Using media to impact health policy-making: an integrative systematic review
Brownson RC, Eyler AA, Harris JK et al. [7]	2018	Getting the Word Out: New Approaches for Disseminating Public Health Science
Brownson RC, Fielding JE, Maylahn CM [17]	2009	Evidence-based public health: a fundamental concept for public health practice
Carroll LN, Au AP, Detwiler LT et al. [18]	2014	Visualization and analytics tools for infectious disease epidemiology: a systematic review
Clar C, Dyakova M, Curtis K et al. [19]	2014	Just telling and selling: current limitations in the use of digital media in public health: a scoping review
Dobbins M, Jack S, Thomas H et al. [20]	2007	Public health decision-makers’ informational needs and preferences for receiving research evidence
Fung IC-H, Tse ZTH, Fu K-W [21]	2015	The use of social media in public health surveillance
Green LW, Ottoson JM, Garcia C et al. [2]	2009	Diffusion theory and knowledge dissemination, utilization, and integration in public health
Nelson DE, Hesse BW, Croyle RT [22]	2009	Making data talk: communicating public health data to the public, policy makers, and the press
Ohlmeier C, Frick J, Prütz F et al. [23]	2014	Nutzungsmöglichkeiten von Routinedaten der Gesetzlichen Krankenversicherung in der Gesundheitsberichterstattung des Bundes
Owen N, Glanz K, Sallis JF et al. [24]	2006	Evidence-based approaches to dissemination and diffusion of physical activity interventions
Richards CL, Iademarco MF, Atkinson D et al. [6]	2017	Advances in Public Health Surveillance and Information Dissemination at the Centers for Disease Control and Prevention
Valdiserri RO, Sullivan PS [25]	2018	Data Visualization Promotes Sound Public Health Practice: The AIDSvu Example
Van Bon-Martens MJH, Achterberg PW, van de Goor IAM et al. [26]	2012	Towards quality criteria for regional public health reporting: concept mapping with Dutch experts
Welch V, Petkovic J, Pardo Pardo J et al. [27]	2016	Interactive social media interventions to promote health equity: an overview of reviews
Wilson PM, Petticrew M, Calnan MW et al. [4]	2010	Disseminating research findings: what should researchers do? A systematic scoping review of conceptual frameworks

The research project ‘Evaluation of National and Regional Public Health Reports’ was conducted within the Health Monitoring Programme of the European Union in 2003. With the aim of identifying quality criteria and best practice models of effective health reporting, national and regional public health reports were collected and analysed. A major conclusion of the project was that it would be beneficial to put more effort in the development of a common methodology for health reporting that provides guidelines at international, national and regional level [9].

The guideline ‘Good Practice in Health Reporting’ has been developed by a working group consisting of representatives from all levels of health reporting in Germany, with the aim of strengthening the field at local, state and national level. The document contains guidelines and recommendations to answer to the need for continual development in health reporting as well as a list of quality criteria that are intended to serve as technical guidance for the preparation of public health reports [10].

These two sources, Eva PHR and the German Good Practice guideline, were studied in depth and analysed for overlaps. Based on the results, categories for quality criteria for public health reports were established, to which the criteria derived from these two sources and from the literature review were assigned. The experts from the InfAct consortium were involved at various stages of the criteria development through informal feedback rounds and discussions: in identifying relevant literature, selecting the criteria, forming the categories, and presenting the results.

## Results

The results of the web-based desk research on health reporting formats and their target groups in EU MS and associated countries are summarised first. In the second section, the quality criteria for public health reports are presented.

### Overview of national health reporting in the EU

The results of the desk research comprise a total of 234 national health reporting formats from 32 countries. For this analysis, each format was only counted once per country. On average over seven different formats are used per analysed country.

**Fig. 1:**
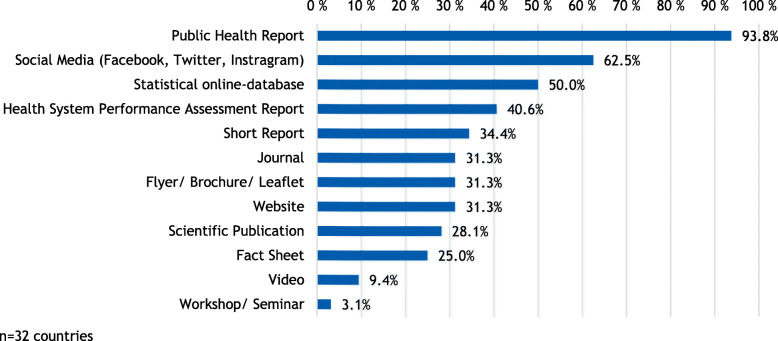
Health reporting formats per country

On country level, public health reports are the most frequently used health reporting format (93,8%), followed by the digital formats social media (62,5%) and statistical online-database (50,0%). Videos (9,4%) and workshops or seminars (3,1%) are the least used communication channels (Fig. 1).

**Fig. 2:**
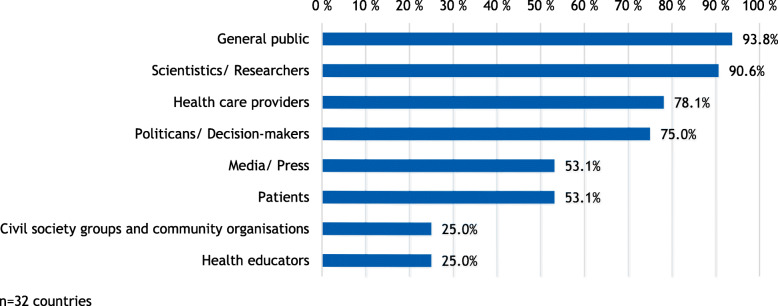
Target groups of health reporting per country

Information on addressed target groups was partially provided within the reporting formats but more often as contextual information on the publishers’ website. Figure 2 shows the stated target groups across reporting formats on country level. The general public (93,8%) as well as scientists and researchers (90,6%) are the most frequently addressed groups. While media and press as well as patients are named as targeted groups in more than half of the countries (53,1%), only a quarter (25,0%) address civil society groups and community organisations as well as health educators.

Table 3 presents the results of the cross-sectional analysis illustrating which target groups are addressed by the formats across countries. 41,4% of public health reports and 33,3% of short reports and journals are addressed to politicians and decision-makers. For the majority of social media (95,0%) and fact sheets (90,9%) as well as all videos and websites, the general public is named as the main target group. Social media is also indicated to disseminate health information to the media and press (85,0%). Patients are only mentioned as a target group by a few formats. Scientists and researchers are particularly addressed by statistical online-databases (87,5%), journals (91,7%) and by workshops and seminars as well as scientific publications. Health educators and health care providers are stated as an important target group by workshops or seminars (100,0%), while health care providers are also mentioned as a major addressee by HSPA reports (76,9%). Civil society groups and community organisations are only stated by a small percentage of the formats, which leads to the generally lower frequency as a target group of health reporting formats.

**Table 3:** Health reporting formats and their stated target groups

	Politicians/ Decision-makers	Health care providers	Scientists/ Researchers	Health educators	Patients	Civil society groups and community organisations	General public	Media/ Press
**Public Health Report**	41,4%	28,8%	51,4%	3,6%	7,2%	6,3%	28,8%	0,9%
**Health System Performance Assessment Report (HSPA)**	100,0%	76,9%	7,7%	7,7%	0,0%	7,7%	7,7%	0,0%
**Short Report**	33,3%	33,3%	26,7%	13,3%	13,3%	0,0%	66,7%	0,0%
**Fact Sheet**	0,0%	18,2%	27,3%	0,0%	27,3%	0,0%	90,9%	0,0%
**Scientific Publication**	11,1%	33,3%	100,0%	0,0%	0,0%	0,0%	11,1%	0,0%
**Journal**	33,3%	41,7%	91,7%	8,3%	0,0%	8,3%	8,3%	0,0%
**Flyer/ Brochure/ Leaflet**	20,0%	20,0%	20,0%	0,0%	30,0%	10,0%	60,0%	10,0%
**Website**	7,7%	0,0%	7,7%	0,0%	30,8%	0,0%	100,0%	0,0%
**Statistical online-database**	25,0%	6,3%	87,5%	0,0%	6,3%	0,0%	25,0%	6,3%
**Workshop/ Seminar**	0,0%	100,0%	100,0%	100,0%	0,0%	0,0%	0,0%	0,0%
**Video**	0,0%	0,0%	0,0%	33,3%	33,3%	0,0%	100,0%	0,0%
**Social Media (Facebook, Twitter, Instagram)**	0,0%	0,0%	5,0%	0,0%	15,0%	5,0%	95,0%	85,0%

Indicates the percentage of formats (left column) addressing a particular target group (upper row) across countries. Since formats usually address several target groups, the row sum can add up to > 100% per format

All in all, the findings of the web-based desk research show a considerable diversity of health reporting practices across EU MS and associated countries.

### Quality criteria for public health reports

**Fig. 3:**
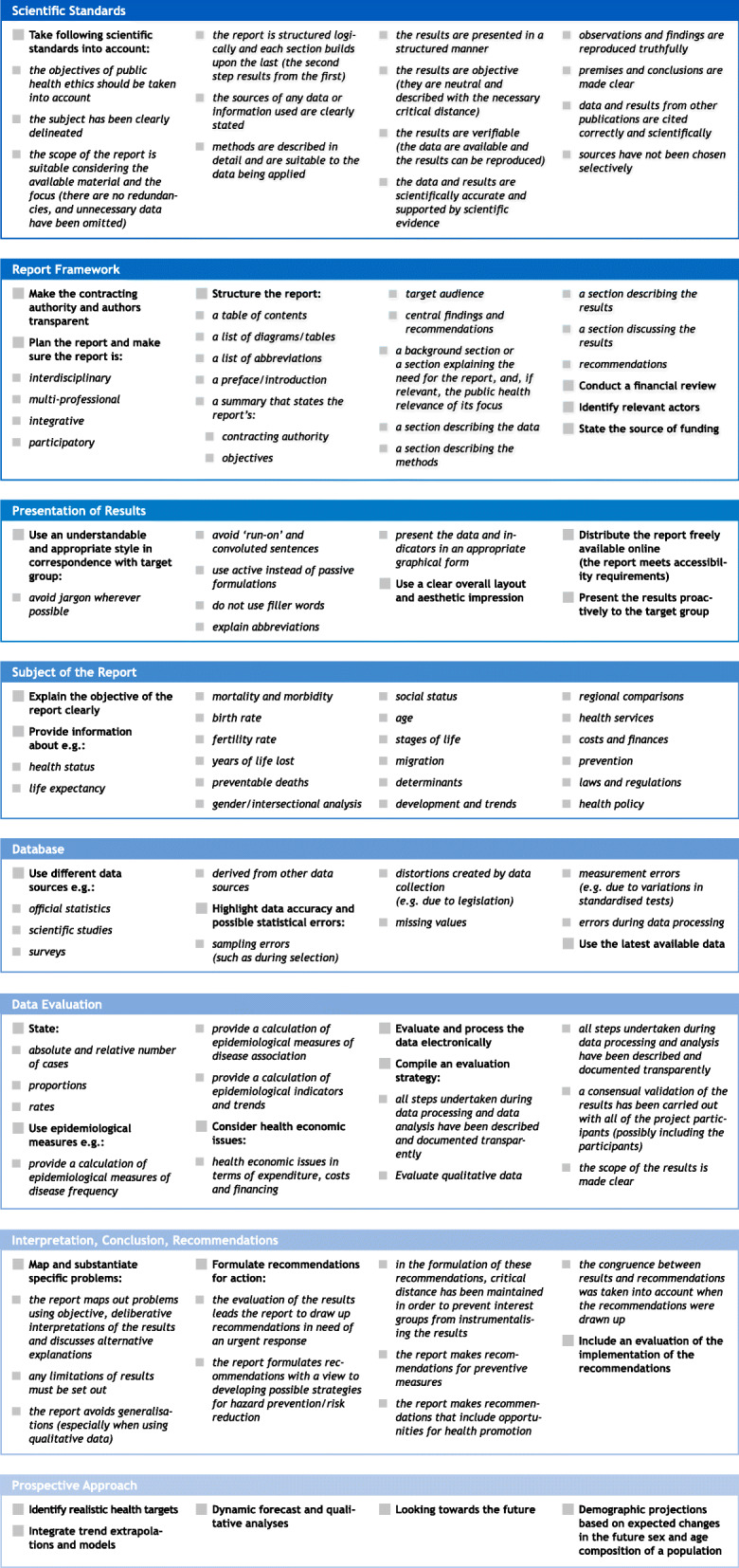
List of quality criteria for public health reports, adapted from [9, 10]

Mainly based on the output of earlier projects and exchange with experts, a number of quality criteria for preparing public health reports could be identified and assigned to eight categories. The results have been compiled in a checklist for easy reference (Fig. 3).

The following paragraphs give an overview of the quality criteria for public health reports by categories.

1. **Scientific standards:** A basic requirement for the preparation of public health reports is compliance with scientific standards. These include a clear definition of topics and a logical structure of the report. In addition, data and results must be scientifically correct and supported by scientific evidence, and observations and findings must be reported truthfully. Furthermore, data and results from other publications must be cited correctly and completely; there should be no selective use of sources.
2. **Framework of the report:** The second category addresses the framework of the report. Authors should ask themselves the questions: Are the target group and authors made transparent? Is the preparation of the report interdisciplinary, multiprofessional, integrative, or participatory in relation to the research question? Does the report follow a defined structure and is funding transparently presented?
3. **Presentation of results:** This category is about the presentation of the results and especially about an understandable, appealing and appropriate style of the report that adequately addresses the defined target groups (e.g., for the general public: easy-to-understand language, avoidance of technical jargon). Other important criteria are the overall layout and free availability in both printed and digital form.
4. **Subject of the report:** The report should contain a comprehensible rationale and description of the report’s objective. Basic considerations should include that the population reported on is correctly represented, the analysis of data is gender-sensitive, the individual social status is considered, changes over time are monitored or regional differences are identified.
5. **Database:** The database category includes criteria for data selection, accuracy and timeliness of data. Data should be selected according to the topic of the report and data should be drawn from a variety of data sources, if possible, to improve the robustness of a result. In addition, potential statistical errors and limitation for example regarding comparability should be considered and the most current data sets available should be used.
6. **Data evaluation:** When analysing data, care should be taken to present a variety of key figures such as numbers of cases, proportions and rates. Proportions, for example, can provide information on the distribution of health-related events, whereas rates provide information on the frequency of health-related events. Adequate use of epidemiologic measures is also important. These include: Measures of disease frequency such as prevalence, incidence, mortality and lethality; measures of disease association such as relative risk, hazard ratio, and odds ratio; and measures of disease impact such as absolute risk difference, relative risk difference and attributable risk. Consideration of health economic issues related to expenditures, costs and financing is also an important component of a public health report. Transparent documentation and description of data preparation and data analysis should be written down in an evaluation strategy and all steps for the analysis of qualitative data should be made transparent.
7. **Interpretation, Conclusion, Recommendations:** This category consists of the quality criteria for interpretation, conclusions and recommendations for action. While the report justifies specific problems, the evaluation of the results leads the report to formulate recommendations for action, as well as an evaluation of the implementation of these recommendations.
8. **Prospective Approach:** To warn of impending health threats or to help identify relevant policy options, demographic projections and dynamic forecasts playing an important role in providing information about future trends. A prospective approach also involves identifying realistic health targets that can be evaluated at a later date.

## Discussion

The findings of the web-based desk research document the heterogeneity of health reporting practices across EU MS and associated countries. Public health reports were identified as the most widely used health reporting format, with scientists and researchers as their most frequently mentioned target groups (51,4%), followed by politicians and decision-makers (41,4%). Patients (7,2%), civil society groups and community organisations (6,3%), health educators (3,6%) as well as media and press (0,9%) are stated in lower proportions. This indicates that, while digitalisation is advancing, public health reports continue to be an important format for disseminating health information. Intending to reach a broad variety of addressees, they are not specifically targeted to politicians and decision makers. This assumption can be supported by the results of the Eva PHR project, pointing out that health reporting is characterised by a high degree of heterogeneity, with most public health reports covering the broadest possible range of health topics and presenting all available data and indicators. In contrast, policy-makers express their need for analysed information on health status and the determinants associated with health care and finances, future health trends and an assessment of the activities undertaken [9]. The fact that some target groups are mentioned to a lower extent as addressees of public health reports could lead to the conclusion that they are either not a priority audience for health reporting practices or that they receive access to health information through other formats and communication channels that seem to be more appropriate for information uptake to empower individuals and communities in attainment of health and wellbeing. Across all reporting formats, the general public is the most frequently addressed target group followed by researchers and scientist. Politicians and decision-makers are named as targeted group in three quarters of the countries.

It is essential that health reporting formats should be tailored to the needs and competencies of the addressed target groups. Furthermore, it is important to reach a wide audience and to share health information in a timely manner. Therefore, the language of scientific communication is important. Using resources efficiently and getting the attention of politicians and decision-makers are important criteria for choosing a suitable communication channel. While the scientific target group is interested in details, understands academic vocabulary and trusts numbers, the non-scientific audience is mainly interested in the key messages and prefers simplified vocabulary. Furthermore, the latter group has a very different understanding of numerical information. The development of a dissemination strategy is fundamental for the communication of results. This includes the identification of target groups, the communication channels to be chosen as well as the use of generally understandable language and attractively and appealingly designed products [5].

In a second step, an overview of quality criteria to be considered in the planning of public health reports was developed. A total of eight categories with a variety of quality criteria for public health reports were identified. The categories range from scientific standards and topic selection to data handling as well as presentation of results. Depending on the topic and complexity of a public health report, the relevance of the quality criteria should be assessed by the authors for their particular case. While the selected quality criteria are mainly derived and merged from best practice models within the Eva PHR project [9] and criteria described in ‘Good Practice in Health Reporting‘for Germany [10], some crucial aspects were emphasised by experts in the field. In this context, ethical principles are particularly important to preserve human dignity and rights. Another highly relevant factor is compliance with data protection regulations. Furthermore, the entire process should be covered by quality assurance, because seriousness and trustworthiness are essential characteristics of health reporting. Quality criteria that could support the uptake of health information for the selected audience and thus may lead to the desired impact would be, for example, adherence to scientific standards, appropriate presentation of results and the formulation of recommendations for action for policy makers. While the presentation of data and indicators in aesthetic graphic form could help to make health information easier to grasp, especially for the non-scientific target group, methodological approaches to the evaluation of data as well as the underlying database could be more relevant for the scientific audience. Using an understandable and appropriate style in correspondence to the target group is a central precondition. All in all, the quality criteria for the preparation of standardised public health reports are transferable to various formats and address general requirements for the dissemination of health information.

During the web-based desk research as well as the process of establishing the quality criteria some challenges have arisen which could limit the findings. In some cases, only national language information was available on the analysed websites. Google translate was used to translate content from these websites in languages other than German or English into English for basic information and preliminary analysis. Furthermore ‘Health reporting’ is not a commonly used terminology in all of the analysed countries. A consistently used definition of health reporting would be helpful to make health information easier to find. Because of inconsistencies in the definition and use, some health reporting formats could only with difficulty be clearly identified and some may have been missed.

This research was conducted before the emergence of the COVID-19 pandemic which has clearly demonstrated the need for rapid response tools to provide health information as quickly as possible. In this situation, dashboards have proven to be an effective health reporting format for presenting and visualising the vast amount of dynamic data. An additional option to disseminate health information could be participatory community listening sessions, which are practiced, for example, in Iceland [28]. However, dashboards, listening sessions as well as policy briefs were not included as possible categories of health reporting formats in the web-based desk research and are therefore missing from the analysis.

Comprehensive conceptual and integrative publications could not be obtained. Interdisciplinary (academic) research would be desirable to investigate in detail which formats and dissemination strategies are most suitable for the requirements of the target groups. Due to this lack of scientific evidence, the quality criteria for the preparation of public health reports could not be compiled for specific target groups. A conceptual approach that incorporates the policy perspective would be useful in order to arrive at conclusions that can advise on policy options and increase the policy impact. An interesting approach was taken by the BAHCI project (‘Bringing a Health Claim to Information’) in the context of the InfAct project which developed a Health Information (HI)-Impact Index to monitor the availability, dissemination and use of evidence by key stakeholders [29].

## Conclusions

For the effective dissemination of health information, it is important to reach a broad audience and to share health information in a timely manner. It is also crucial to consider in advance which target groups the reporting is aimed at and to choose an adequate reporting format accordingly. To this end, it would be desirable to define quality criteria for every health reporting format, in order to tailor the information in the best possible way to the needs and competencies of the targeted groups including criteria for appropriateness of information uptake. This requires further research, preferably in an interdisciplinary approach.

Our work aimed to facilitate the harmonisation of health reporting practices across the EU while reducing health information inequalities. It should be seen as a step towards better access to health information that follows a set of relevant standards for improved quality and comparability. Integrating the findings into health reporting training programmes could foster capacity building and practical training in applying the recommendations as well as increase the reach. In addition to health reporting formats, it would be desirable to harmonise the definitions of indicators within organisations and countries to reduce the burdens on health reporting.

The quality criteria for public health reports presented here are included in a guidance document that also contains general recommendations for national health reporting, making it a useful tool for other health reporting formats as well [30]. The findings are applicable at regional, national as well as international level and could be integrated into a future European health information infrastructure, like the envisaged Distributed Infrastructure on Population Health (DIPoH) [31], to enhance sustainability.

## Data Availability

Please contact the corresponding author for data requests.

## Abbreviations

DIPoH: Distributed Infrastructure on Population Health
EU: European Union
HI: Health Information
HIS: Health Information System
HSPA: Health System Performance Assessment
IANPHI: International Association of National Public Health Institutes
InfAct: Information for Action - Joint Action on Health Information
MS: Member States
WHO: World Health Organization
